# Gestational and Breastfeeding Low-Protein Intake on Blood Pressure, Kidney Structure, and Renal Function in Male Rat Offspring in Adulthood

**DOI:** 10.3389/fphys.2021.658431

**Published:** 2021-04-22

**Authors:** Gabriela Leme Lamana, Ana Leticia Luchiari Ferrari, José Antonio Rocha Gontijo, Patrícia Aline Boer

**Affiliations:** Fetal Programming and Hydroelectrolyte Metabolism Laboratory, Nucleus of Medicine and Experimental Surgery, Department of Internal Medicine, Faculty of Medical Sciences at State University of Campinas, Campinas, Brazil

**Keywords:** fetal programming, low-protein diet, lactation, epithelial-mesenchymal transition, tubular kidney dysfunction, arterial hypertension

## Abstract

**Background**: Our previous studies demonstrated that maternal protein-restricted (low-protein, LP) 16-week-old offspring had pronounced nephron number reduction and arterial hypertension associated with an unchanged glomerular filtration rate (GFR). An enhanced gomerular area may be related to increased glomerular filtration and overflow, which accounts for glomerular filtration barrier breakdown and early glomerulosclerosis. The effect of protein restriction during gestational and breastfeeding periods is unknown.

**Method**: The functional e-structural kidney evaluation was obtained using lithium and creatinine clearance, kidney morphometry, immunoblotting, and immunostaining analysis in 16 and 24-week-old LP offspring compared to age-matched NP progeny.

**Results**: Low protein rats’ progeny had significantly reduced birth weight, without previous catch-up growth phenomena, in parallel with a decreased adiposity index. Transforming growth factor-beta 1 (TGF-β1) glomerular expression was significantly enhanced in the LP group. Also, the LP offspring had a 38% lower nephron number and an increased glomerular volume. They also presented with a higher cardiac index and arterial blood pressure compared with age-matched NP offspring. The LP rats exhibited augmented Na+/K+-ATPase in the proximal segments, and NOS1 immunoreactivity in whole renal tissue was associated with sodium retention in the proximal nephron segments. We also found significantly enhanced collagen content associated with increased TGFβ1 and ZEB1/2 renal immunoreactivity in LP offspring compared with NP offspring. Increased hypertrophy markers in LP podocytes were associated with an amplified IL-6/STAT3 pathway activity.

**Conclusion**: To our knowledge, these are the first data demonstrating renal functional and structural changes in protein restriction during gestation and lactation model of fetal programming. The fetal-programmed adult offspring showed pronounced structural glomerular disorders with an accentuated and advanced fibrosis stage, without a change in the GFR. These findings suggest that the glomerular enhanced TGF-β1 action may induce ZEB1/2 expression that may cause glomeruli epithelial-to-mesenchymal transition. Besides, decreased nephron number in the LP offspring with preserved glomerular function may be related to protective or even attenuate the activated IL-6/STAT3 pathway.

## Introduction

Fetal programming by gestational low-protein intake results in reduced birth weight, 28% fewer nephrons, reduced renal salt excretion, and increased risk of cardiovascular and renal disorders in adults (Schreuder et al., 2006; Mesquita et al., 2010a,b; Sene et al., 2013, 2018; Vaccari et al., 2015). Despite the unchanged glomerular filtration rate (GFR), these results were associated with a 17% increment in the glomerular tuft area. Previous studies revealed that protein and caloric intake restriction in rats results in embryonic/fetal growth retardation (IUGR), accompanied by impaired nephron development (Mesquita et al., 2010a,b). The tubular dysfunction associated with enhanced sodium and water reabsorption might, at least in part, be responsible for the programming of adult arterial hypertension. Recent studies on programmed progeny by maternal protein restriction also show that enhanced sympathetic neural activity may involve increased tubular salt and water transport disorders (Vaccari et al., 2015; Custódio et al., 2017). Kidney disorders in maternal protein-restricted offspring may partly be secondary to reduced nephron numbers associated with glomeruli overflow, basal membrane breakdown, and early glomerulosclerosis (Sene et al., 2013, 2018). The lack of nutrients may result in pivotal pathway signaling changes during several stages of fetal development, which, in turn, may cause irreversible organ and system disorders in adulthood (Barker, 1995, 2003; Langley-Evans, 2006, 2009). In rats, nephron development involves a tight control of gene expression, protein synthesis, tissue remodeling, and different compensative challenges to maintain homeostasis from birth to adult life. After delivery, the programmed rats’ day-to-day adaptations to maintain body salt and water homeostasis may involve changes in signaling molecular cell pathways’ expression. As such, the present study aimed to determine how severe protein restriction during pregnancy and breastfeeding alters kidney function and structure in 16- and 24-week-old male offspring. We studied the blood pressure and tubular sodium handling, evaluated by lithium clearance, in conscious maternal protein-undernourished rats compared with their appropriate healthy maternal food intake controls.

Thus, environmental and genetic interactions interfere with ontogenic cell proliferation and differentiation, leading to structural and dysfunctional organ development effects in distinct organs. The epithelium-mesenchymal transition (EMT) is a critical adaptive phenomenon characterized by the interchangeable cell plasticity process between the epithelial and mesenchymal phenotypic states (Liu, 2004; Kalluri and Weinberg, 2009). These phenotypic changes are accompanied by a reduction in the genetic expression of transcription factors epithelial markers associated with the high expression of mesenchymal markers, such as desmin and nestin (Kalluri and Weinberg, 2009). The evolution of chronic kidney damage is accompanied by irreversible renal parenchymal fibrosis. In this pathophysiological process of renal fibrosis, there is a fundamental involvement of EMT as the typical final route that leads to chronic kidney disease (CKD; Liu, 2010). The transforming growth factor-beta (TGF-β) triggers tubular EMT, and its expression is regulated positively in several types of CKDs (Chung et al., 2010). In isolated glomeruli, we found that adult male children of mothers fed a protein-restricted diet during pregnancy showed reduced miR-200f expression associated with increased expression of TGF-β1 and ZEB2 for glomerular EMT and expression of glomerular fibrosis markers (Sene et al., 2013). Besides, studies have shown that IL-6 plays a crucial role in regulating various physiological and pathological processes. IL-6 is stimulated by the JAK/STAT3 signal transduction cascade, which leads to nuclear phospho-STAT3 translocation, which is implicated in cell growth, proliferation, differentiation, and survival (Postigo and Dean, 2000). Despite several changes observed in the maternal adult LP offspring’s renal structure and function, the renal functional and structural patterns in the progeny restricted to proteins during pregnancy and breastfeeding remain unknown. The current study evaluates whether maternal protein intake restriction during whole pregnancy and breastfeeding alters kidney functional and morphological response beyond 16-week of life in male rats. Therefore, we also verified the renal expression of protein markers of fibrosis and inflammation during the severe restriction of protein maternal and breastfeeding ingestion periods in adult offspring compared with appropriate controls.

## Materials and Methods

### Experimental Animals

The experiments were conducted on age-matched Wistar Hannover rats (0.250–0.300 kg), which were allowed free access to water and standard rat chow. The Institutional Ethics Committee on the Use of Animals (CEUA/UNICAMP) approved the experimental protocol according to the general guidelines established by the Brazilian College of Animal Experimentation (COBEA), and the NIH Guide for the Care and Use of Laboratory Animals was followed throughout the study (protocol number #4242-1). Our local colonies originated from a breeding stock supplied by CEMIB/Unicamp, Campinas, SP, Brazil. Immediately after weaning at 3 weeks of age, the animals were maintained under controlled temperatures (25°C) and lighting conditions (7:00–19:00), with free access to tap water and standard rodent laboratory chow (Nuvital, Curitiba, PR, Brazil) and followed up to 12 weeks of age. The animals were mated using the permanent polygamous system; the day that sperm were seen in the vaginal smear was designated as day 1 of pregnancy. The dams were singly caged and randomly assigned into two groups: daily food supply of regular protein diet (NP, 17% protein) or low-protein diet (LP, 6% protein) chow ad libitum throughout pregnancy and lactation. The NP and LP maternal food consumption were determined daily and later normalized for body weight, also recorded daily in both groups. The NP and LP maternal food consumption were determined daily (subsequently normalized for body weight), and the bodyweight of dams was recorded weekly in both groups. The dams’ body weight and food consumption were determined daily and weekly (subsequently normalized for body weight). Male pups from the NP and LP litters were weighed at birth. Post-weaning was followed and maintained with normoprotein standard rodent laboratory chow weekly until the 24th week of life. The NP and LP offspring were euthanized and sampled at 16 and 24 weeks of age. For sampling, a cohort of pups was euthanized at 16- and 24-week-old with a mixture of ketamine (75 mg kg^−1^ body weight, i.p.) and xylazine (10 mg kg^−1^ body weight, i.p.), and the level of anesthesia was controlled by corneal reflex monitoring. The animals (*n* = 60) were then perfused *via* transcardiac perfusion with saline containing heparin (5%) for 15 min under constant pressure, followed by 0.1 M phosphate-buffered saline (PBS, pH 7.4) containing 4% (w/v) paraformaldehyde and 0.1 M sucrose for 25 min. After perfusion, the kidneys were removed, weighed, and representative samples were fixed in 4% phosphate-buffered formalin for 24 h for paraffin embedding. The retroperitoneal and perigonadal adipose tissues were collected from the 24-week-old NP, and LP offspring (*n* = 49) after 5% isoflurane inhalation anesthesia was administered. The adiposity index was calculated as the ratio of total adipose tissue weight to body weight. The kidneys were excised, weighed, and tissue samples were collected for histology, immunohistochemistry, and immunoblotting tests.

### Blood Pressure Measurement

The systolic blood pressure (SBP) of the NP offspring (*n* = 18–20) and LP (*n* = 12–20) was measured from 8 to 24 weeks in conscious rats using an indirect tail-cuff method. Blood pressure was assessed using an electrosphygmomanometer (BP-2000 Blood Pressure Analysis System, Visitech Systems, Austin, TX, United States) combined with a pneumatic pulse transducer/amplifier. This indirect approach allowed repeated measurements with a close correlation (correlation coefficient = 0.975) compared with direct intra-arterial recording. The mean of 10 consecutive SBP readings represented the measured blood pressure. Measurements were performed under controlled temperature and luminosity at the same time during the day. The double product (DP) was calculated as the heart rate product (HR) × SBP, and the units for HR and blood pressure were beats per minute and mmHg, respectively.

### Renal Function Test

Renal function was tested on the last day at 24 weeks of age in awake and unrestrained NP (*n* = 15) and LP (*n* = 11) male offspring. Renal clearance of creatinine (CCr) and lithium (CLi) was calculated using the standard formula (C = UV/P) based on urinary and plasma creatinine and lithium levels. CCr was used to estimate GFR, and CLi+ was used to assess proximal tubule output. Fractional urinary sodium excretion (FENa+) and fractional potassium excretion (FEK+) were calculated as CNa+/CCr and CK+/CCr, respectively. Fractional proximal sodium excretion (FEPNa +) and fractional post-proximal sodium excretion (FEPPNa +) were calculated as CLi+/CCr × 100 and CNa+/CLi+ × 100, respectively (Mesquita et al., 2010a; Vaccari et al., 2015; Custódio et al., 2017). Plasma and urinary sodium, potassium, and lithium concentrations were measured using flame photometry (B262; Micronal, São Paulo, Brazil). Creatinine was determined spectrophotometrically (362; Micronal, São Paulo, Brazil) using the alkaline picrate method. The results are reported as mean ± SD per 100 g body weight.

### Morphometric Analysis and Stereological Estimation of Kidney Volume, Glomerular Number, and Glomerular Volume

Renal stereology estimates the glomerular volume, and the total number of renal corpuscles per kidney was performed in 12-day old offspring using the combination of dissector/fractionator technique as described by Mandarim-de-Lacerda (2003; Erratum in 2007). Total offspring body and kidney masses were determined for both the NP (*n* = 5) and LP (*n* = 5) offspring obtained from different litters. We used the fractionator method to estimate the number of glomeruli. Briefly, NP and LP offspring were anesthetized (ketamine, 75 mg kg^−1^ body weight, and xylazine 10 mg kg^−1^ body weight, i.p.). The left kidney was removed, weighed, and the Cavalieri principle estimated the volume. Each group’s kidney was sliced longitudinally in two halves; this preliminary procedure, using one-half kidney, was used as a pilot to determine the average diameter of glomeruli in the subsequent studies. The material was fixed, included in paraplast, the entire kidney was exhaustively sliced into serial sections of 5 μm, and ordered sequentially. The 4-mm-slices were processed and stained with hematoxylin-eosin, analyzed in an Olympus® microscope, photographed, and posteriorly submitted to study by the Image-Pro Express 6.0® software. The glomerular diameter was evaluated by taking kidney sections passing through the macula densa, and the average of glomeruli diameters was obtained for each experimental group. The necessary spacing kidney section was established, considering the prior glomeruli diameter study. In another kidney half portion, the isotropic samples from NP and LP groups were obtained through two consecutive cuts, where the first cut was made at a random angle. Then, the cut kidney surface was sectioned again at a random angle. In this way, we could admit that the obtained fragment presented tissue in an isotropic form. The material was then processed and sliced according to the spacing previously determined in the pilot diameter study for its respective group (NP: 23 cuts dispensed/LP: 28 cuts issued). The histological renal slices were stained with hematoxylin-eosin, and glomeruli were counted. The total number of glomeruli was calculated as: N (gl) = N (count) × fractionation, where fractionation = kidney weight (g) /slice weight (g).

The cortex’s volume was determined. The material was photographed in 100x magnification using a grid system of central points of squares; a count was made, taking into account how many points pass through the renal cortex. The cortex’s volume was then obtained, calculated as the sum of these points, multiplied by the cut’s thickness (5 μm) So, Vcortex = ∑A × spacing × 5 μm.

The total volume of glomeruli [V(gl)] was then determined using the same software. A dot mask was inserted in glomeruli photos, and the number of points that touched the glomeruli was counted. Thus, VV(gl) = Pp/Pt where, Pp = points counted and Pt = total points, such as V (gl) = VV(gl) × Vcortex.

Additionally, the total glomerular volume [V(gl)] was calculated using the average glomerular volume times glomeruli number by the followed equation: V (average gl) = V (gl) × N (gl), where N is the number of glomeruli.

### Immunohistochemistry Study: Tissue Processing, Histology, and Immunohistochemical Procedures

Twenty-four-week-old NP (*n* = 5) and LP (*n* = 5) male offspring were anesthetized with 5% isoflurane inhalation. The level of anesthesia was controlled by corneal reflex monitoring. The animals were then perfused with saline containing heparin (5%) for 15 min under constant pressure, followed by 0.1 M phosphate buffer (pH 7.4) containing 4% (w/v) paraformaldehyde and 0.1 M sucrose for 25 min. After perfusion, the kidneys were removed, weighed, and representative samples were fixed in 4% phosphate-buffered formalin for 24 h for paraffin embedding. The paraffin blocks were cut into 5 μm-thick sections. For immunohistochemical analysis, paraffin sections were incubated overnight at 4°C with primary antibodies for anti-Na/K-ATPase (Santa Cruz Biotechnology, Inc., CA, United States, 1:100 dilution), anti-TGF-β1 (Santa Cruz Biotech, Inc., CA, United States, 1:250 dilution), anti-desmin (Santa Cruz Biotech, Inc., CA, United States, 1: 200 dilution), anti-nestin (Santa Cruz Biotech, Inc., CA, United States, 1: 600 dilution), anti-collagen I (Sigma-Aldrich Co., St. Louis, MO, United States, 1:400 dilution), and anti-ZEB1 (Santa Cruz Biotech, Inc., CA, United States, 1:400 dilution). Secondary antibodies were used following the primary antibody host. Proteins were immunohistochemically detected using the avidin-biotin-peroxidase method. Briefly, 5 μm-thick deparaffinized kidney sections on poly-L-lysine-coated slides were treated with 3% H2O2 in PBS for 15 min, nonfat milk for 60 min, primary antibodies for 60 min, and avidin-biotin-peroxidase solution (Vector Laboratories Inc., CA, United States, 1:50 dilution). The kidney sections were finally stained with 3,3'-diaminobenzidine tetrahydrochloride substrate (Sigma-Aldrich Co., St. Louis, MO, United States) to demonstrate the sites of peroxidase binding. The slides were counterstained with Harris’ hematoxylin.

Five cortical and medullar fields of each histological section were analyzed, and the average immunoreactivity reading was determined. Images were captured with a photomicroscope and analyzed using Leica Qwin 3.1 for Windows. No immunoreactivity was observed in the control experiments in which one of the primary antibodies was omitted.

### Immunoblotting

Twenty-four-week-old NP (*n* = 5) and LP (*n* = 5) male offspring were anesthetized with 5% isoflurane inhalation. Anesthesia was controlled by monitoring the corneal reflex, and the animal’s abdominal cavity was opened for kidney removal. The tissue was minced coarsely and homogenized immediately in 10 volumes of solubilization buffer [10 ml/L Triton-X 100, 100 mM Tris(hydroxymethyl)amino-methane (Tris) pH 7.4, 10 mM sodium pyrophosphate, 100 mM sodium fluoride, 10 mM ethylenediaminetetraacetic acid, 10 mM sodium vanadate, 2 mM phenylmethylsulfonyl fluoride, and 0.1 mg/ml of aprotinin] at 4°C, using a polytron PTA 20S generator (model PT 10/35, Brinkmann Instruments, Westbury, NY, United States) operated at maximum speed for 20 s. The tissue extracts were centrifuged at 11,000 rpm at 4°C for 40 min, and the supernatants were used as samples. Protein quantitation was performed using the Bradford method. The tissue extract samples (250 μg of protein) were subjected to sodium dodecyl sulfate-polyacrylamide gel (SDS-PAGE) electrophoresis. After electrophoretic separation, proteins were transferred to nitrocellulose membranes and blotted with a specific antibody. The samples were treated with Laemmli buffer containing 100 mM dithiothreitol, heated in a boiling water bath for 4 min, and subjected to 8% SDS-PAGE in a Bio-Rad mini gel apparatus (Mini-Protean, Bio-Rad). The protein was transferred from the gel to the nitrocellulose membranes for 90 min at 120 V (constant) using a Bio-Rad miniature transfer apparatus (Mini-Protean). Nonspecific protein binding to the nitrocellulose was reduced by pre-incubation at 22°C for 2 h in a blocking buffer (5% nonfat dry milk, 10 mM Tris, 150 mM NaCl, and 0.02% Tween 20). Nitrocellulose blots were incubated at 4°C overnight with primary antibodies diluted in a blocking buffer (3% nonfat dry milk, 10 mM Tris, 150 mM NaCl, and 0.02% Tween 20) as follows: AT1R (Santa Cruz Biotech, Inc., CA, United States, 1:1000 dilution), AT2R (Santa Cruz Biotech, Inc., CA, United States, 1:1000), JAK2 (Santa Cruz Biotech, Inc., CA, United States, 1:1000 dilution), STAT3 (Santa Cruz Biotech, Inc., CA, United States, 1:1000 dilution) NOS1 (Santa Cruz Biotech, Inc., CA, United States, 1:1000 dilution), NOS2 (Santa Cruz Biotech, Inc., CA, United States, 1:1000 dilution), SOD2 (Santa Cruz Biotech, Inc., CA, United States, 1:1000 dilution), IL-6 (Santa Cruz Biotech, Inc., CA, United States, 1:1000 dilution), pJAK2 (Cell Signaling Technology, Inc., MA, United States, 1:1000 dilution), pSTAT3 (Cell Signaling Technology, Inc., MA, United States, 1:1000 dilution), ERK1/ERK2 (Cell Signaling Technology, Inc., MA, United States, 1:1000 dilution), and β-actin (Cell Signaling Technology, Inc., MA, United States, 1:1000 dilution). Membranes were stained with Coomassie Brilliant Blue dye before blotting to ensure equal protein loading. All membranes were incubated with β-actin antibodies to avoid possible inconsistencies in protein loading and transfer. Only homogeneously stained membranes were used in this study. Immunoreactive bands were detected using the chemiluminescence method (SuperSignal West Pico Chemiluminescent Substrate, Thermo Scientific, United States). Images of the developed radiographs were scanned (HP DeskJet Ink Advantage 4625), and the band intensities were quantified using optical densitometry (Scion Image Corporation).

### Data Presentation and Statistical Analysis

All data are reported as mean ± SD. Data obtained over time were analyzed using one-way ANOVA. *Post hoc* comparisons between means were performed using Bonferroni’s contrast test when one-way ANOVA analysis indicated statistical differences between groups. Comparisons between the two groups throughout the weeks were performed using two-way ANOVA for repeated measurements. The first factor was the protein content in the pregnant dam’s diet, and the second factor was time. When an interaction was significant, the mean values were compared using Tukey’s posthoc analysis. Student’s t-test evaluated studies involving only two independent samples, within or between groups. The Brown-Forsythe and Welch’s tests and ANOVA tests were used to correct situations characterized by heteroscedasticity (different variances between groups). GraphPad Prism was used for data analysis (GraphPad 8.0 Software, Inc., La Jolla, CA, United States). The level of significance was set at *p* ≤ 0.05.

## Results

### Body Mass, Adiposity Index, and Kidney Morphometry

**Figure 1:**
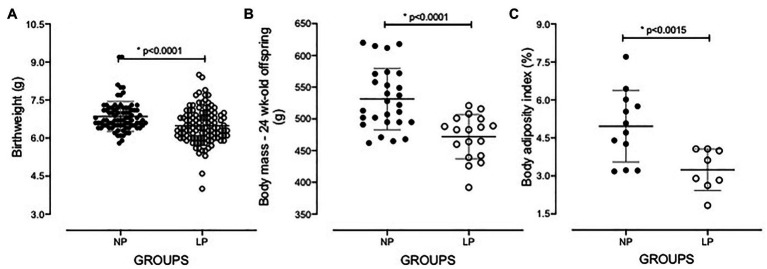
The low-protein (LP) and NP birthweight (**A**; LP: *n* = 117; NP: *n* = 84), and body weight (**B**; LP: *n* = 18; NP: *n* = 27) and body adiposity index (**C**; LP: 18; NP: *n* = 27), in grams, of 24-week-old protein-restricted (LP) progeny compared with age-matched standard (NP) progeny. Data are presented as scatter dot plots; Welch’s *t*-test was used for data analysis. The level of significance was set at *p* < 0.05.

**Figure 2:**
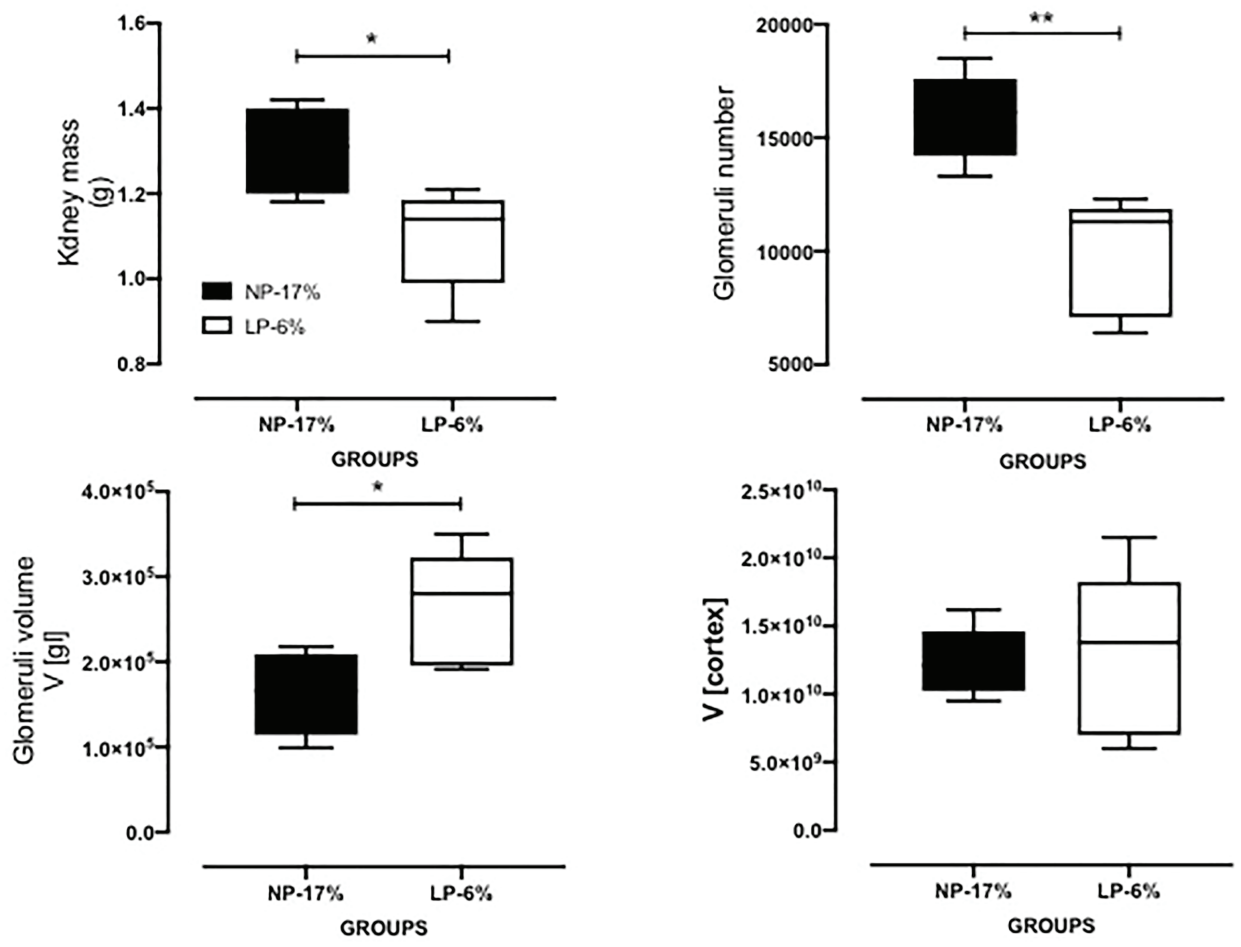
The kidney mass (in grams), glomeruli number and volume, and cortical volume of 24-week-old LP progeny (*n* = 5) compared to age-matched NP offspring (*n* = 5). Data are presented as scatter dot plots; unpaired Welch’s *t*-test was used for data analysis. The level of significance was set at ^*^p=0.01; ^**^p=0.0015.

Gestational and breastfeeding protein restriction intake did not significantly change the body mass of pregnant dams (NP: 278.5 ± 6.239 g, *n* = 24; LP: 266.5 ± 3.794 g, *n* = 39). Besides, it did not affect the number of offspring (NP: 9.7 ± 3, *n* = 178; LP: 8.6 ± 4, *n* = 246; *p* = 0.28) and the proportion of male and female offspring (*p* = 0.3245) per litter. The birth weight of LP male pups (body mass relative to that of the entire litter; *n* = 117) was significantly reduced compared with that of NP pups (*n* = 84; NP: 6.854 ± 0.5928 g vs. LP: 6.491 ± 0.6820 g; *p* = 0.0001; Figure 1). The body mass of 24-week-old LP offspring (471.9 ± 34.91 g, *n* = 18) remained lower than that of age-matched NP progeny (531.3 ± 48.49 g, *n* = 27, *p* = 0.0001). It was associated with a reduced adiposity index in the LP offspring (3.242 ± 0.8214) relative to the NP group (4.962 ± 1.413; *p* = 0.0015; Figure 1). Additionally, at 16 weeks after birth, both the left and right kidney masses of LP offspring (1.098 ± 0.1201 g, *n* = 5) were significantly reduced compared with that of age-matched NP offspring (1.302 ± 0.1021 g, *n* = 5, *p* = 0.0100; Figure 2).

**Figure 3:**
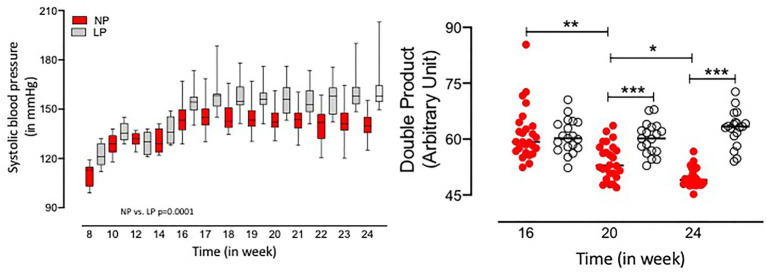
Systolic blood pressure (SBP; in mmHg) and double product (DP) index (in arbitrary unit) from 8 to 24 weeks of age in LP (*n* = 12–20) progeny compared to age-matched NP offspring (*n* = 18–20). Data are presented as boxes and whiskers (minimum and maximum values); Welch’s ANOVA test was used for data analysis. The level of significance was set at ^*^p = 0.0256; ^**^p = 0.0117; ^***^p = 0.001.

The present study evaluated the glomerular number in 16-week-old LP offspring kidney compared to age-matched controls by stereological analyses. In gestational and breastfeeding protein-restricted animals, a significant reduction in the number of glomeruli was observed in the LP progeny [about 36%; LP (*n* = 5): 9850 ± 2587; NP (*n* = 5): 15.940 ± 1926; *p* = 0.0015, Figure 2]. Simultaneously, an increase in glomerular volume accompanied by a reduced nephron number in the LP group compared with the control group [LP (*n* = 5): 263.270 ± 67.108; NP (*n* = 5): 162.600 ± 49.017; *p* = 0.0134, Figure 2] was observed with no change in renal cortex volume (*p* = 0.4352). As shown in Figure 3, the SBP increased progressively, being significant from the 17th to 24th weeks of age (17th week of age, LP: 157.4 ± 8.2 mmHg vs. NP: 146.1 ± 8.6 mmHg, *p* < 0.02; 24th week of age, LP: 164.4 ± 13.4 mmHg vs. NP: 139 ± 7.0 mmHg, *p* = 0.001). A higher calculated DP accompanied the increment in blood pressure in LP (*p* = 0.001) than in NP progeny (Figure 3).

### Renal Function Data

**Figure 4:**
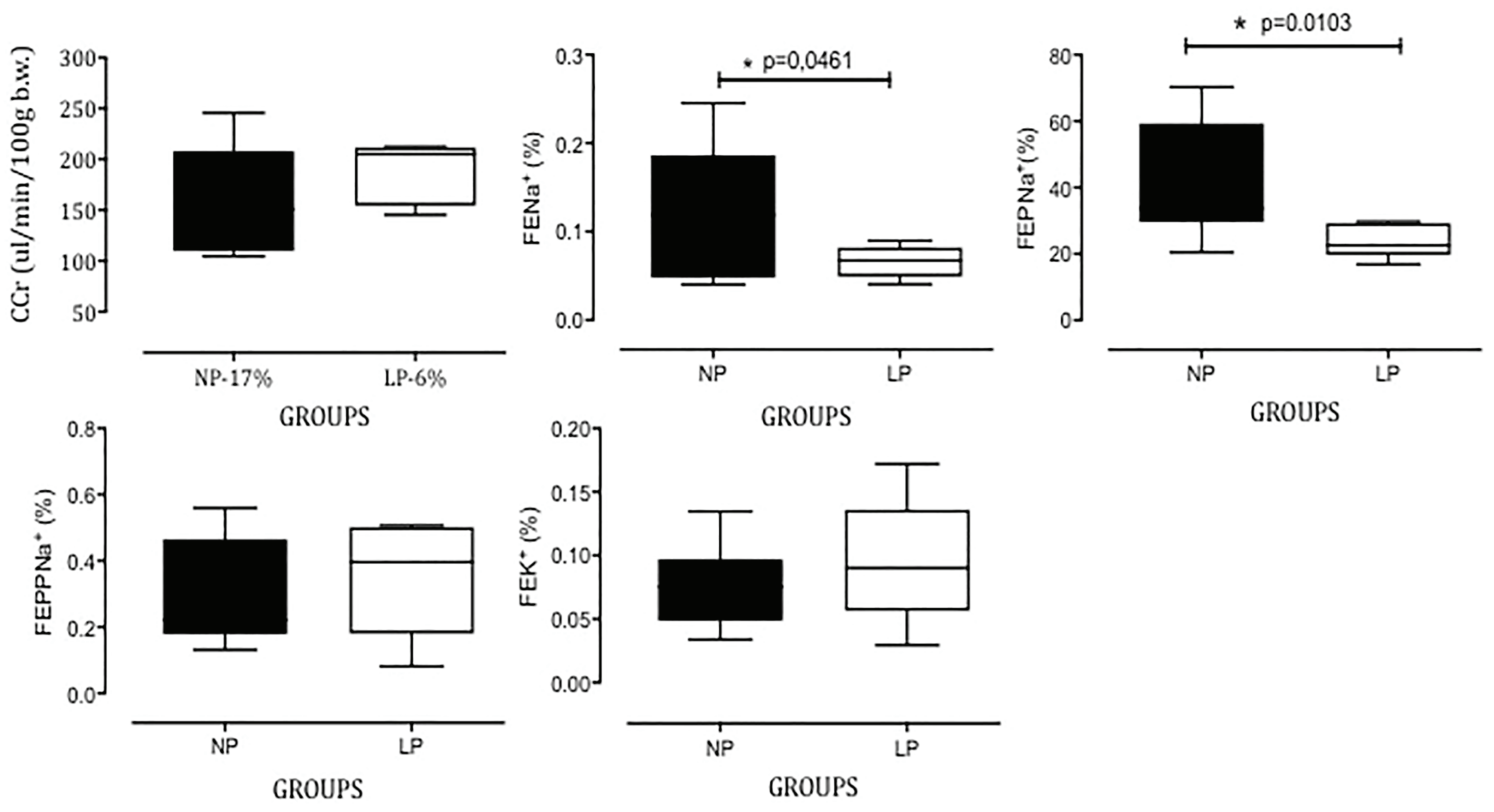
Renal function; creatinine clearance (*C*Cr), fractional sodium excretion (FENa+), proximal (FEPNa+), and post-proximal (FEPPNa+) fractional sodium excretion, and fractional potassium excretion (FEK+) in 16-week-old LP offspring (*n* = 11) compared to age-matched NP progeny (*n* = 15). Data are presented as boxes and whiskers (minimum and maximum values); unpaired Welch’s *t*-test was used for data analysis. The level of significance was set at *p* < 0.05.

The data for renal function performed on the last day at 24 weeks of age in male offspring of both the NP and LP groups are summarized in Figure 4. The plasma sodium (LP: 147 ± 4.8 mM vs. NP: 144 ± 4.5 mM), plasma potassium (LP: 4.7 ± 0.9 mM vs. NP: 4.5 ± 0.9 mM), plasma lithium (LP: 86 ± 19 mM vs. NP: 78 ± 23 mM), urinary flow rates and glomerular filtration rates, estimated by CCr, did not significantly differ between the groups during the renal tubule sodium handling studies (LP: 187.5 ± 30.85 μl/min/100 g body weight (b.w.) vs. NP: 157.4 ± 56.15 μl/min/100 g b.w., *p* = 0.2103). FENa+ was significantly lower in the LP offspring (0.06407 ± 0.018%, *n* = 7) than in the NP age-matched offspring (0.1186 ± 0.077%, *n* = 9; *p* = 0.0461). The decreased FENa+ occurred in parallel with a significant reduction in FEPNa+ (LP: 24.13 ± 5.031% vs. NP: 41.84 ± 17.29%, *p* = 0.0103; Figure 4). The decreased FENa+ in the LP progeny was also accompanied by unchanged FEPPNa+ (LP: 0.2918 ± 0.1584% vs. NP: 0.3475 ± 0.1656%, *p* = 0.2527) and FEK+ (LP: 0.9794 ± 0.4357% vs. NP: 0.7464 ± 0.2981%, *p* = 0.0534) compared with the NP age-paired control group (Figure 4).

### Immunohistochemistry and Immunoblotting Analyses

**Figure 5:**
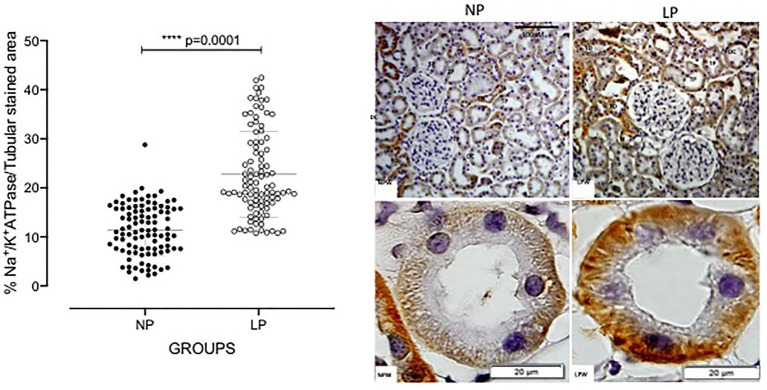
Kidney Na/K-ATPase expression by immunohistochemistry in 24-week-old LP progeny (*n* = 5) compared with age-matched NP progeny (*n* = 5). A magnification of 400× and 1000× was used. The scale bar can be found at the bottom right of each figure. Five cortical and medullar fields of each histological section were analyzed, and the average immunoreactivity reading was determined. Data are presented as scatter dot plots on the left; unpaired Welch’s t-test was used for data analysis. The level of significance was set at *p* < 0.05.

**Figure 6:**
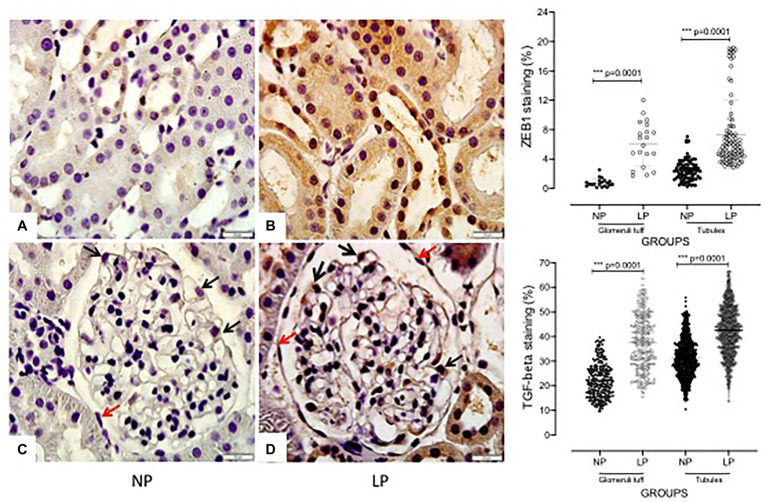
Glomerular and tubular TGFβ1 and ZEB1 immunoperoxidase labeling in 24-week-old male NP offspring (**A**/**C**; *n* = 5) compared with age-matched LP progeny (**B**/**D**; *n* = 5). A red arrow indicates perivascular epithelial cell (PEC); a black arrow characterizes podocyte. A 400× and 1000× magnification was used in the tubular area **(A,B)** and glomerular area **(C,D)**, respectively. The scale bar can be found at the bottom right of each figure. Five cortical and medullar fields of each histological section were analyzed, and the average immunoreactivity reading was determined. Data are presented as scatter dot plots on the right; the unpaired Welch’ ANOVA test was used for data analysis. The level of significance was set at *p* < 0.05.

The current study showed that the Na/K-ATPase expression in the basolateral membrane tubule proximal segments of the 24-week-old LP (Figure 5) considerably increased compared with that of the age-matched NP. In the 24-week-old LP offspring, TGF-β1 was slightly expressed in the glomerular basement membrane delimiting the glomerular capillaries, as well as in the proximal tubule (Figure 6). The current study also demonstrated an increased glomerular TGF-β1 immunostain and the basal portion of the proximal and post-proximal tubular cells.

**Figure 7:**
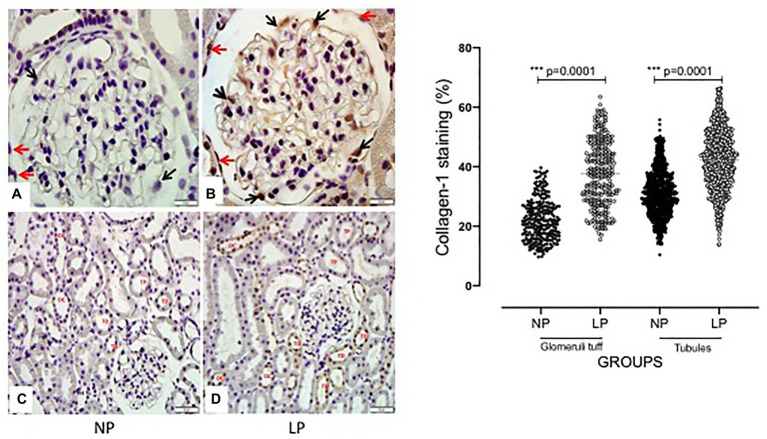
Glomerular and tubular collagen-1 immunoperoxidase staining in 24-week-old male NP offspring (*n* = 5; **A**/**C**) compared with age-matched LP progeny (*n* = 5; **B**/**D**). A 400× and 1000× magnification was used in the tubular area **(A,B)** and glomerular area **(C,D)**, respectively. The scale bar can be found at the bottom right of each figure. Five cortical and medullar fields of each histological section were analyzed, and the average immunoreactivity reading was determined. Data are presented as scatter dot plots on the right; the unpaired Welch’ ANOVA test was used for data analysis. The level of significance was set at *p* < 0.05.

**Figure 8:**
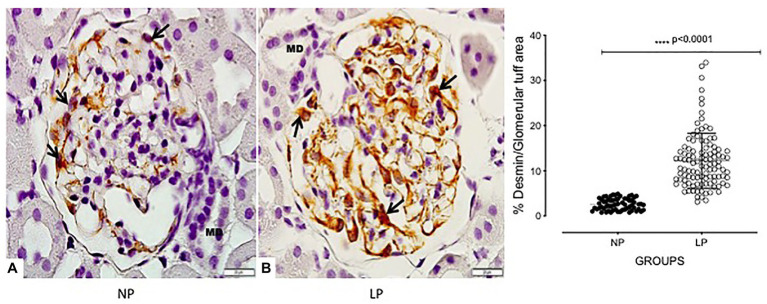
Glomerular tuff desmin immunoperoxidase staining in 24-week-old male NP offspring (*n* = 5; **A**) compared with age-matched LP progeny (*n* = 5; **B**). The black arrow indicates podocytes, and the *macula densa* is shown as MD. A 1000× magnification was used in the glomerular area. The scale bar can be found at the bottom right of each figure. Five cortical and medullar fields of each histological section were analyzed, and the average immunoreactivity reading was determined. Data are presented as scatter dot plots on the right; unpaired Welch’s *t*-test was used for data analysis. The level of significance was set at *p* < 0.05.

**Figure 9:**
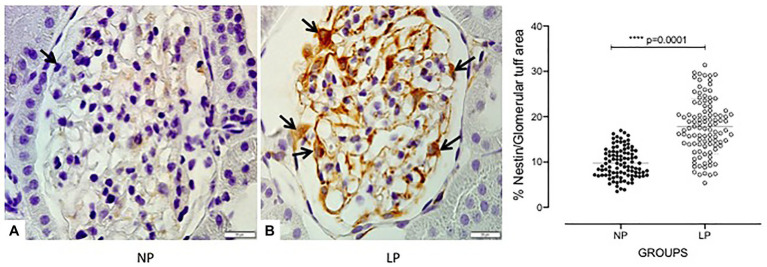
Glomerular tuff nestin immunoperoxidase staining in 24-week-old male NP offspring (*n* = 5; **A**) compared with age-matched LP progeny (*n* = 5; **B**). The black arrow indicates podocytes. A 1000X magnification was used in the glomerular area. The scale bar can be found at the bottom right of each figure. Five cortical and medullar fields of each histological section were analyzed, and the average immunoreactivity reading was determined. Data are presented as scatter dot plots on the right; unpaired Welch’s *t*-test was used for data analysis. The level of significance was set at *p* < 0.05.

**Figure 10:**
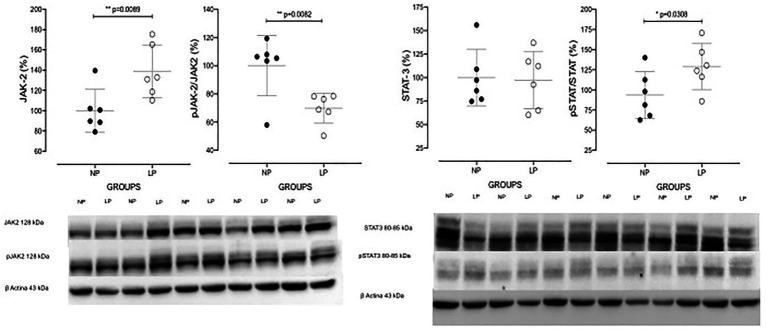
Western blot analyses of JAK-2, pJAK-2, STAT-3, and pSTAT-3 protein expression in the whole kidneys of 24-week-old NP (*n* = 5) and LP (*n* = 5) offspring. Data are presented as scatter dot plots; unpaired Welch’s *t*-test was used for data analysis. The level of significance was set at *p* < 0.05.

**Figure 11:**
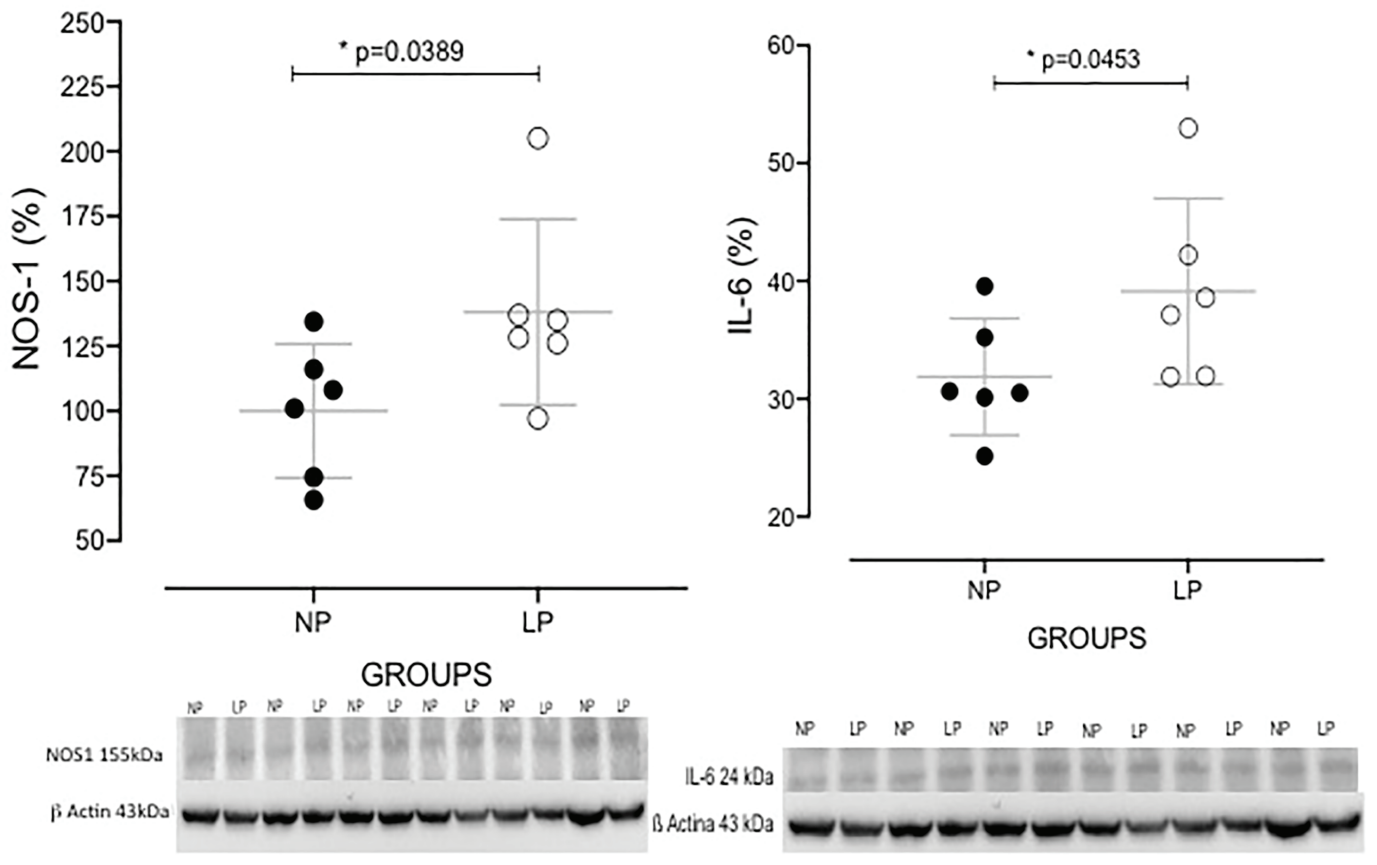
Western blot analyses of NOS1 and IL-6 protein expression in the whole kidneys of 24-week-old NP (*n* = 5) and LP (*n* = 5) offspring. Data are presented as scatter dot plots; unpaired Welch’s *t*-test was used for data analysis. The level of significance was set at *p* < 0.05.

The increased TGF-β1 immunomarker was associated with high ZEB1 staining in LP rats compared with NP rats of the same age (Figure 6). The findings also indicated a high and extensive tubular and glomerular type I collagen immunoreactivity in the LP offspring relative to the NP offspring (Figure 7). A higher desmin and nestin immunoreactivity was observed in the glomeruli podocyte and renal tubules of 24-week-old LP rats compared to age-matched NP progeny (Figures 8, 9). Significant enhancement of both JAK-2 and pSTAT-3 levels (Dot plots chart) were also verified in the whole kidney of 24-week-old LP rats through Western blotting. It was associated with reduced pJAK-2 and unchanged STAT-3 levels (Figure 10) compared with the NP age-matched offspring. Additionally, NOS1 and IL-6 expression was enhanced in the whole kidney of the 24-week-old LP offspring compared with that of the NP offspring (Figure 11). No changes were observed in NOS2, SOD, and GH immunoblotting analyses.

## Discussion

The role of the kidney in the genesis of arterial hypertension has long been established. Brenner et al. (1998) proposed that some individuals are susceptible to hypertension and renal injury because of reduced nephron number. A reduction in nephron number and, therefore, in the whole kidney glomerular filtration area would result in decreased salt excretory capacity, enhanced susceptibility to hypertension, and reduced renal reserve upon excessive sodium intake (Schor et al., 1980). Embryonic and fetal protein restriction may lead to a long-term disorder in renal sodium and water handling and structural changes that may be associated with arterial hypertension onset in male adult progeny (Mesquita et al., 2010a,b; Sene et al., 2013, 2018; Vaccari et al., 2015). Therefore, the limiting compensation for renal injury may, at least in part, explain the higher prevalence of hypertension and renal disease observed in populations with a high prevalence of low birth weight (Barker, 1995, 2003; Lackland et al., 2000, 2002; Hsu et al., 2003). However, despite several kidney morphological and functional disorders in the maternal restricted-protein intake model, the effect of protein restriction on gestational and breastfeeding low-protein ingestion (LP) remains unknown. Herein, we focused on hypertension development as an outcome and its association with nephron number and structure changes and reduced urinary sodium excretion in LP offspring. It is essential to state that sex hormones determine sexual phenotype dimorphism in the fetal-programmed disease model in adulthood by changes in the long-term control of neural, cardiac, and endocrine functions. Thus, the present study was performed in male rats considering the findings above to eliminate interferences due to gender differences (Kwong et al., 2000; Gillette et al., 2017). The current study confirmed that low birth weight (approximately 13%) might reflect the influence of inappropriate protein intake during pregnancy and lactation on embryonic/fetal growth and development. Unpredictably, LP offspring’s body mass, beyond the second week of age, remained lower than that of age-matched NP progeny during the allover follow-up. The phenomenon known as catch-up growth was not observed in this model, unlike the observed mass recovery observed in mothers’ offspring subjected to protein restriction supply only during the gestational period. Although the placenta is capable of self-regulation, the compensatory mechanism does not seem sufficient in protein restriction cases, especially in the final pregnancy period, where fetal growth is predominant and accelerated (Fowden et al., 2006). A previous study in rodent fetal programming models demonstrated that impairment in placental transport of nutrients, mainly involving amino acids, may be related, at least in part, to the decline in placental vascularization (Burdge and Lillycrop, 2010). Barker (1995) presented the theory that the maternal environment could induce adult diseases based on observed epidemiological associations between low birth weight and an increased risk for ischemic heart diseases and, particularly, arterial hypertension. Although it seemed that the relationship is not invariant, the bulk of evidence suggested a meaningful direct or indirect interaction between low birth weight and subsequent hypertension (Barker, 1995, 2003). As such, our results confirmed the findings of previous studies, which demonstrated a marked and sustained rise in arterial blood pressure associated with a decreased urinary sodium excretion in low-birth-weight LP compared with that in age-matched NP offspring (Mesquita et al., 2010a,b). However, unlike previous findings in the gestational protein-restricted model that demonstrated an enhanced arterial pressure beyond 7 weeks of age in LP relative to NP counterparts, the present study extended the protein restriction throughout lactation and revealed a consistent onset delay of hypertension at adulthood. The reduced glomeruli number in this model, including protein intake reduction and breastfeeding, was higher than observed previously for us. A 36% reduction in nephron number was also confirmed in the gestational and breastfeeding protein-restricted model compared with a 27% reduction when restriction occurred only during the gestational period (Mesquita et al., 2010a,b; Sene et al., 2013). Additionally, a pronounced decline in FENa+ was observed in the 24-week-old maternal protein-restricted male offspring. This effect was accompanied by intensive sodium reabsorption in the nephron’s proximal segments despite unchanged CCr, FEK+, and post-proximal sodium handle FEPPNa+ (Figure 4). The present study confirmed previous findings from our laboratory, demonstrating that even when associated with decreased nephron number units, maternal food-restricted offspring maintain normal whole GFR estimated by creatinine clearance. Herein, a reduced remaining glomerular number was associated with an increased glomerular volume, probably by enhancing glomerular volume with compensatory enhanced blood flow and glomerular hyperfiltration despite a loss of efficiency on the filter barrier (Mesquita et al., 2010a,b; Sene et al., 2013, 2018; Vaccari et al., 2015). The renal results and the increased blood pressure in the maternal low protein diet intake model corroborated hypothesis of Brenner et al. (1998). Hyperfiltration in low birth weight leads to subsequent glomerular hypertension, resulting in sustained renal function disorder. Once the glomerular filtration rate does not change significantly due to protein restriction in LP offspring, compensatory poorly adapted nephron functional changes may have occurred intrinsically when nephrogenesis was compromised. A previous study suggested that the late-onset arterial hypertension induced by maternal low-protein intake is associated, at least in part, with an increased renal neural activity. An explanation for a reduced renal sodium excretion maybe explain by decreasing the proximal tubule sodium rejection, associated with unchanged creatinine clearance, and sodium was usually filtered (Custódio et al., 2017). In the present study, the persistent and enhanced DP in the LP progeny strongly suggested an increased sympathetic activity in the LP group relative to the control group.

The reduced urinary sodium excretion is significantly attenuated by renal denervation (Custódio et al., 2017). Experimental studies have supported the hypothesis that fetal programming correlates with the deregulation of sodium transporters in different nephron segments (Manning et al., 2002; Alwasel and Ashton, 2009; Baum, 2010). To the best of our knowledge, the present data demonstrated for the first time that a decreased urinary sodium excretion in gestational and breastfeeding LP offspring was associated with a reduction in proximal tubular sodium reabsorption that was incompletely compensated by distal nephron segments. do Carmo Pinho et al. (2003), in a study of renal function and Na+ transporters in male rats born to low-protein diet dams, showed that the transcriptional upregulation of Na+ transport could have contributed to hypertension. Interestingly, by Western blot analysis, the present study found that Na/K-ATPase protein was upregulated in the low-protein diet male progeny compared with the standard diet intake offspring (Figure 5). Considering a study showing that AngII AT2R is an effective inhibitor of Na/K-ATPase (De Souza et al., 2004; Alwasel and Ashton, 2009), we previously demonstrated that the downregulation of the renin-angiotensin system, found in 16-week-old LP male offspring, might also explain the lack of inhibition of Na/K-ATPase (Mesquita) in this model. The finding may support the results obtained here by western blot analysis and the low sodium excretion rate because Na/K-ATPase participates in Na+ reabsorption in the basolateral membrane of the proximal segments of the nephron (Figure 5). Fekete et al. (2008) demonstrated that AngII promotes Na/K-ATPase-α1 subunit translocation from the cytoskeletal fraction into the cytosol under experimental conditions, which must be taken into account. Prolonged elevated glomerular filtration and blood flow processes may cause the reduced nephron number manifested as an accelerated renal function loss and glomerulosclerosis. These findings were highly prevalent among adult LP offspring with low birth weight (Mesquita et al., 2010a,b; Sene et al., 2013). Glomeruli overflow may be an initial insult that initiates a cascade of events, including an early inflammatory phase followed by a fibrotic response. Irreversible renal fibrosis is a common consequence of most renal injuries (Liu, 2010; Zeisberg and Neilson, 2010). TGF-β regulates extracellular matrix protein deposition in renal tissue (Lee, 2012). To our knowledge, these are the first data showing renal morphological and protein expression changes in the gestational and lactation protein restriction model of fetal programming. In the current study, the protein-restricted fetal-programmed adult rats showed structural glomerular and tubular disorders with remarkable TGF-β1 and collagen-1 immunoreactivity, suggesting an advanced fibrosis stage. These findings led us to speculate that the renal TGF-β1 upregulation may induce ZEB 2 expression, which may cause glomerular EMT as evidenced by increased nestin and desmin expression.

Epithelium-mesenchymal transition is associated with fibrosis progression (Carew et al., 2012). Studies have implicated TGF-β1 and ZEB2 overexpression with the phenotypic changes during EMT in progressive renal development of fibrosis (Kato et al., 2007; Wang et al., 2010, 2011). The glomerular corpuscle is composed of different mesenchymal and epithelial cell types, and the EMT process may be uneven throughout the renal parenchyma. Four resident cell types constitute the renal corpuscles: mesangial cells, endothelial cells, visceral cells (podocytes), parietal epithelial cells (PECs) with specific glomerular functions and protein expressions. Podocytes are terminally differentiated epithelial cells with a low proliferation capacity (Kriz, 1996); thus, injury and loss of these cells can lead to glomerular scarring (Liu, 2010; Zeisberg and Neilson, 2010). The present study verified strikingly enhanced glomerular and tubular expressions of TGF-β1 and type I collagen, intrinsically related to the fibrotic process, by immunohistochemistry. We hypothesized that the hemodynamic glomerular overload in the LP progeny compared with the NP progeny associated with an enhanced blood flow in the remaining nephrons stimulated fibrous processes by enhancing TGF-β1 collagen 1. In parallel, an altered cellular corpuscles phenotype to EMT occurred by expressing mesenchymal markers, namely nestin and desmin. Thus, earlier findings indicated that glomerular podocytes might undergo phenotypic conversion, characterized by the loss of podocyte-specific markers and gain of transitional features, a process reminiscent of EMT (Li et al., 2008). TGF-β triggers EMT, and its expression is upregulated in virtually every type of chronic kidney disease (Yang and Liu, 2001; Bottinger and Bitzer, 2002), including in the diseased LP programmed model.

In the present study, we speculated that EMT, a process induced by TGF-β1 and ZEB 2 overexpression, plays a significant role in imparting PEC plasticity. PEC plasticity results in non-differentiated progenitor cell glomerular migration, an early and ruling response of nephron cells in the LP renal pathological process. Glomerular insults are potent inducers for the generation of IL6 by the tubular epithelial cells functioning as a cross-talk between the glomerulus and the tubule (Su et al., 2017). IL-6 may induce type-I collagen by tubular epithelial cells and accelerate tubulointerstitial fibrosis, which has been associated with increased STAT3 phosphorylation (Nechemia-Arbely et al., 2008; Ranganathan et al., 2013). This study observed a significant increase in IL-6 (23%) and the phosphorylated fraction of STAT3 (28%) in the LP progeny compared with the age-matched NP progeny. Although JAK2 increased by 40%, the phosphorylated JAK fraction decreased by 23%. Thus, we assumed that the high expression of JAK might be responsible for the phosphorylation of STAT3. Renal cells, including podocytes, endothelial cells, mesangial cells, and tubular epithelial cells, secrete IL-6. Thus, IL6 over-reactivity may induce mesangial dysfunction, increase cell proliferation, increase mesangial matrix deposition, and glomerular sclerosis (Coletta and Polentarutti, 2000; Gohda et al., 2001; Lu, 2008; Nechemia-Arbely et al., 2008). Studies suggested that fetal programming and chronic kidney disease in adult offspring are associated with increased maternal ROS production and high oxidative stress in fetal tissues (Halliwell, 2013; de Brito Alves et al., 2016). The present study did not demonstrate significant renal changes in the SOD and ROS levels. However, we observed an unprecedented increase of 32% in the NOS1 content in the renal of 24-week-old LP, relating to the kidney tubular O_2_ consumption in parallel with increased sodium and water reabsorption stimulation the proximal tubule. This effect, particularly in the proximal segments of nephrons, was associated with a determining factor, which was the basolateral expression of the Na/K-ATPase pump. As observed in the laboratory LP model (Mesquita et al., 2010b), our study revealed a quantitative increase in Na/K-ATPase in the 24th week of life in LP offspring. The elevated Na/K-ATPase in the 24-week-old LP offspring’s proximal tubules may involve an increase in NOS1 expression associated with increased reabsorption of sodium and water.

In conclusion, it is plausible to deduce an association between accentuated reduction of the nephron numbers, decreased natriuresis, and reciprocal changes in tubular Na/K-ATPase with the development of arterial hypertension found in severe gestational and breastfeeding protein-restricted progeny compared with age-matched NP rats. After birth, the overload of an economic kidney may result in an increased glomerular overflow. This overload in fetal-programmed rats can result in pronounced structural glomerular disorders and accentuated and advanced stage of fibrosis promoted by TGF-β1 action inducing ZEB 2 expression, which may cause premature nephron senescence in parallel with functional loss.

## Data Availability Statement

The raw data supporting the conclusions of this article will be made available by the authors, without undue reservation.

## Ethics Statement

The animal study was reviewed and approved by Committee on the Use of Animals (CEUA/UNICAMP) according to COBEA (protocol number #4242-1).

## Author Contributions

GL: data curation, investigation, formal analysis, methodology, visualization, and writing–original draft. AF: methodology. JG: formal analysis, methodology, visualization, and writing–review and editing. PB: conceptualization, formal analysis, funding acquisition, methodology, resources, supervision, visualization, writing–original draft, and writing–review and editing. All authors contributed to the article and approved the submitted version.

### Conflict of Interest

The authors declare that the research was conducted in the absence of any commercial or financial relationships that could be construed as a potential conflict of interest.
